# Multifaceted Intervention to Improve Graft Outcome Disparities in African American Kidney Transplants (MITIGAAT Study): Protocol for a Randomized Controlled Trial

**DOI:** 10.2196/57784

**Published:** 2024-10-10

**Authors:** Morgan Overstreet, Hannah Culpepper, Deanna DeHoff, Mulugeta Gebregziabher, Maria Aurora Posadas Salas, Zemin Su, Jessica Chandler, Felicia Bartlett, Paige Dunton, Taylor Carcella, David Taber

**Affiliations:** 1 Medical University of South Carolina Charleston, SC United States

**Keywords:** kidney transplant, mobile health, medication adherence, mHealth, nephrology, transplant surgery, postoperative monitoring, telemedicine, eHealth

## Abstract

**Background:**

The outcome disparities for African American recipients of kidney transplant is a public health issue that has plagued the field of transplant since its inception. Based on national data, African American recipients have nearly twice the risk of graft loss at 5 years after transplant, when compared with White recipients. Evidence demonstrates that medication nonadherence and high tacrolimus variability substantially impact graft outcomes and racial disparities, most notably late (>2 years) after the transplant. Nonadherence is a leading cause of graft loss. Prospective multicenter data demonstrate that one-third of all graft loss are directly attributed to nonadherence. We have spent 10 years of focused research to develop a comprehensive model explaining the predominant risk factors leading to disparities in African American kidney recipients. However, there are still gaps in patient-level data that hinder the deeper understanding of the disparities. Lack of data from the patient often lead to provider biases, which will be addressed with this intervention. Culturally competent, pharmacist-led interventions in medication therapy management will also address therapeutic inertia. Pharmacist interventions will mitigate medication access barriers as well (cost and insurance denials). Thus, this multidimensional intervention addresses patient, provider, and structural factors that drive racial disparities in African American kidney recipients.

**Objective:**

This prospective, randomized controlled trial aimed to determine the impact of multimodal health services intervention on health outcomes disparities in African American recipients of kidney transplant. The aims of this study are to improve adherence and control of late clinical issues, which are predominant factors for racial disparities in kidney recipients, through a technology-enabled, telehealth-delivered, 4-level intervention.

**Methods:**

The Multifaceted Intervention to Improve Graft Outcome Disparities in African American Kidney Transplants (MITIGAAT) study is a 24-month, 2-arm, single-center (Medical University of South Carolina), 1:1 randomized controlled trial involving 190 participants (95 in each arm), measuring the impact on adherence and control of late clinical issues for racial disparities in kidney recipients, through a technology-enabled, telehealth-delivered, 4-level intervention. The key clinical issues for this study include tacrolimus variability, blood pressure, and glucose control (in those with diabetes mellitus). We will also assess the impact of the intervention on health care use (hospitalizations and emergency department visits) and conduct a cost-benefit analysis. Finally, we will assess the impact of the intervention on acute rejection and graft survival rates as compared with a large contemporary national cohort.

**Results:**

This study was funded in July 2023. Enrolled began in April 2024 and is expected to be complete in 2026. All patients will complete the study by the end of 2028.

**Conclusions:**

In this protocol, we describe the study design, methods, aims, and outcome measures that will be used in the ongoing MITIGAAT clinical trials.

**Trial Registration:**

ClinicalTrials.gov NCT06023615; https://www.clinicaltrials.gov/study/NCT06023615

**International Registered Report Identifier (IRRID):**

PRR1-10.2196/57784

## Introduction

### Background

Racial disparities in kidney transplants is a public health issue that has persisted for more than 50 years. As compared with White kidney recipients, African American kidney recipients have nearly twice the risk of graft loss at 5 years after transplant. Despite recent data demonstrating modest improvements in this disparity, a kidney transplanted today is still expected to function about half as long in African American people [1-3]. Marginal improvements in African American recipients are due to improved access to transplants, stemming from organ allocation policy changes and reductions in early acute rejection through a better understanding of immunological risk and the use of potent immunosuppression [4-6]. However, recent studies from our team and others demonstrate that posttransplant outcome disparities in African American people can predominately be explained by issues that arise late (≥2 years) after transplant [7-9]. These issues include late medication nonadherence leading to high tacrolimus variability and rejection and poor control of diabetes and hypertension [7-13]. In models adjusting for these late issues, the risk of graft loss in African American recipients was reduced by up to 75%, as compared with non–African American recipients. [7-11].

For the past decade, our research team has focused on improving posttransplant outcomes and reducing disparities in African American kidney recipients by developing and using mobile health (mHealth) and telehealth interventions [14-20]. Our mHealth app and intervention are founded on the self-determination theory and are designed to improve patient autonomy, competence, and relatedness [21-23]. This is coupled with the use of pharmacist-led motivational interviewing (MI) delivered during televisits, which is designed to improve care and identify and address barriers preventing optimal adherence and comorbidity management, including medication costs [24-27]. Our mHealth-based telehealth intervention improves access to care through remote patient monitoring and medication therapy management, addressing structural barriers, racial bias, and therapeutic inertia that induce disparities [28-30]. Through robust preliminary research, we developed, refined, and tested the functionality, acceptance, and durability of our intervention [14-17]. We completed a 60-patient (39/60, 65% African American) pilot study (National Institute of Diabetes and Digestive and Kidney Diseases [NIDDK] K23DK099440), demonstrating significant improvements in the control of diabetes and hypertension through our mHealth intervention; improvements in hypertension control were more substantial in African American recipients [16]. In 2020, we completed a 12-month randomized controlled trial (RCT; Agency for Healthcare Research and Quality [AHRQ] R18HS023754) in 136 patients (87/136, 64% African American) that used our mHealth, remote patient monitoring, pharmacist-led telehealth intervention to improve medication adherence and reduce hospital readmissions and costs by over 40% [14,20]. These 2 clinical trials demonstrated that our intervention is highly accepted in African American people; 55% of our kidney transplant population is African American, while 65% (88/136) of study participants were African American. Dropout rates were 3% [16,20]. An economic analysis demonstrated that the intervention provided a net cost savings of US $368,839 through reduced hospitalizations and had a return of investment of US $4.30 for every dollar spent [31].

### Objectives

The next clear step in our research trajectory is to conduct a long-term, large-scale study and assess whether this 4-component health services intervention can improve health outcome disparities in African American recipients of kidney transplant. Thus, we propose an RCT to use this system to deliberately address posttransplant disparities in African American people entitled the “Multifaceted Intervention to Improve Graft Outcome Disparities in African American Kidney Transplants (MITIGAAT)” study. The overarching hypothesis for the MITIGAAT study is that late nonadherence and suboptimal control of diabetes and hypertension are more common in African American recipients of kidney transplant and are major contributors to health disparities. A multimodal intervention that addresses these issues will significantly reduce health care disparities among African American recipients of kidney transplant.

## Methods

### Study Design

This is a single-center, 24-month, 2-arm, 1:1 RCT involving 190 participants (95 in each arm), with roughly 55% (100 participants) being African American. All patients will be recruited from the Medical University of South Carolina (MUSC), in Charleston, South Carolina (ClinicalTrial.gov NCT06023615). This study was funded by the National Institute of Health’s (NIH) NIDDK. We will also conduct a retrospective longitudinal cohort study, comparing a large contemporary national cohort of adult veteran kidney recipients transplanted between 2015 and 2021 with our RCT intervention arm. This study meets all guidelines set by ClinicalTrials.gov and has been approved by the local Institutional Review Board. The statistician conducting the analyses will be blinded to the treatment arm (semiblind). The randomization will occur through computer-generated random numbers, with no stratification, in blocks of 10. REDCap (Research Electronic Data Capture; Vanderbilt University) will be used for computerized randomization and arm assignment, which will be done by a study team member.

### Aims

The primary outcome of this study is to determine the impact of multilevel health services intervention on achieving improved adherence to tacrolimus. In addition, we will determine the impact of multilevel health services intervention on blood pressure (BP) and glucose control (in those with diabetes). We will conduct a cost-benefit analysis, assessing the estimated costs of hospitalizations and emergency department (ED) visits compared with the cost to deliver interventions. Finally, we will track the incidence of acute rejection, graft loss, and death in the intervention patients; compare our data with a large national cohort of veteran patients of kidney transplant; and assess for racial disparities for these health outcomes. We will enroll both African American and non–African American people to allow us to directly compare these groups across treatment arms to assess disparities.

### Recruitment, Screening, and Enrollment Procedures

Patients will be recruited from the MUSC kidney transplant roster. Adult (≥18 years old at the time of transplant) recipients of kidney transplant having a posttransplant period of more than 2 years who meet the eligibility criteria will be approached by researchers for consideration of participation. After discussing the details of the study, providing time, and gaining informed consent, patients will be randomized in a 1:1 fashion (blocks of 10) to either the enhanced control group (usual care and attention control) or the intervention group (usual care and 4-level intervention [Table 1]). For the aim-4 cohort study, we will include all adult solitary kidney recipients transplanted between 2015 and 2021, with more than 2 years of follow-up, to match the RCT. Complete discussion of this follow-up cohort study is beyond the scope of this RCT methodology paper.

**Table 1:** Overview of the objectives for each visit at each timepoint.

Months and telehealth visit number		Techniques used and topics covered
**1-2**		
	1	Teach patient how to use app, home BP^a^, and glucometer
	2	Review technology with patient, troubleshoot any issues
**3-4**		
	3	Using MI^b^, discuss current control of hypertension and barriers to optimal control
	4	Educate on importance of hypertension control and implement strategies to improve self-monitoring and management
**5-6**		
	5	Using MI, discuss current control of DM^c^ and barriers to optimal control
	6	Educate on importance of DM control and implement strategies to improve self-monitoring and management
**7-8**		
	7	Using MI, discuss current control of anemia and barriers to optimal control
	8	Educate on importance of anemia control and implement strategies to improve management
**9-10**		
	9	Using MI, discuss adherence to immunosuppression and barriers to optimal adherence
	10	Educate on importance of medication adherence and implement strategies to optimize adherence
**11-12**		
	11	Educate importance of staying active and implement strategies to improve staying active and exercising
	12	Educate importance of smart dietary choices and implement strategies to choose wisely
**13-14**		
	13	Review current hypertension control and using MI, identify barriers to optimal control
	14	Implement additional strategies to improve hypertension control
**15-16**		
	15	Review current DM control and using MI, identify barriers to optimal control
	16	Implement additional strategies to improve DM control
**17-18**		
	17	Review current anemia control and using MI, identify barriers to optimal control
	18	Implement additional strategies to improve anemia control
**19-20**		
	19	Review medication adherence and implement strategies to improve if not optimal
**21-22**		
	20	Discuss any ongoing issues with patient precluding optimal management of comorbidities and adherence
**23-24**		
	21	Study close-out session. Conduct surveys and implement plan for continued engagement

^a^BP: blood pressure.

^b^MI: motivational interviewing.

^c^DM: diabetes mellitus.

### Eligibility

#### Inclusion

Participants must be adult (≥18 years of age at the time of enrollment) recipients of kidney transplant at least 2 years after their kidney transplant.

#### Exclusion

We will exclude patients who are recipients of multiorgan transplants or those patients who received a transplant of an organ that is not a kidney (liver, lung, heart, intestine, pancreas, and bone marrow). Any patients who are not capable of measuring their own BP and glucose (if applicable); using mHealth apps after adequate training; and speaking, reading, and hearing English will be excluded.

### Data Capture and Management

Data capture will be accomplished using electronic extraction, manual chart abstraction, and through direct patient interviews (when necessary) by a study coordinator. Data will be collected by conducting a thorough review of the patient’s medical records with patient phone calls at 3-month intervals. Data collection will include all baseline donor or recipient demographics and transplant characteristics as well as medications, laboratory data, pathology, and clinical events that occur during the study. Data will also be captured through the web-based portal and linked to abstracted data within the research database. The REDCap system will be used for data management. REDCap is a secure, web-based application designed exclusively to support data capture for research studies, which we have used in our previous clinical trials. REDCap provides (1) an intuitive interface for data entry (with data validation); (2) audit trails for tracking data manipulation and export procedures; (3) automated export procedures for seamless data downloads to common statistical packages (SPSS [IBM], SAS [SAS Institute], Stata [StataCorp], and R [R Development Core Team]); (4) procedures for importing data from external sources; and (5) advanced features, such as branching logic and calculated fields.

### Sample Size and Power

This study is powered to detect clinically meaningful and statistically significant improvements in medication adherence (aim 1), systolic BP (SBP) control (aim 2), diabetes mellitus (DM) control (aim 3), and graft survival (aim 4) while also demonstrating significant reductions in African American disparities for these end points (overarching aim). The analyses take full advantage of all measurements captured during the entire 2-year study using repeated measures methodology. For tacrolimus variability (aim 1), there will be 9 assessments (baseline and every 3 months), aligning with when laboratories are measured. Over the 2-year study, a 5% trajectory difference between arms for tacrolimus variability is considered clinically meaningful and is feasibly achievable given our pilot data (time×treatment). Tacrolimus concentration coefficient of variation (tac CV) has an expected SD of 8.5 with an approximate Gaussian distribution; thus, meeting normality assumptions. A sample size of 87 per group (N=174) achieves >0.999 power, assuming both arms start with a tac CV of 35% and change over the 2-year study to 32% in the control arm and 27% in the treatment arm. The study is powered at 0.800 to specifically test for reductions in racial disparities between the intervention and control arms, using a 3-way interaction term in the model (time×treatment×race). For BP and glucose control (aim 2), we will capture all home- and ambulatory-based measures aggregated into monthly assessments, totaling 25 (baseline and once monthly for 24 periods). We have 0.998 power to detect a difference in SBP changes between arms, assuming a mean SBP of 135 mm Hg in both arms at baseline and a 5 mm Hg reduction in the treatment arm, using 25 repeated measures (monthly). SBP has an expected SD of 9 with a Gaussian distribution; thus, meeting normality assumptions. We have 0.800 power to test for reductions in racial disparities between the arms for SBP, using the time×treatment×race 3-way interaction term. We have 0.998 power to detect a difference between arms for glucose changes, assuming a mean baseline glucose of 160 mg/dL in both arms, 50% (87/174) of patients having DM at the time of randomization, and a trajectory difference in glucose of 24 mg/dL between arms (25 repeated measures). We have 0.803 power to test for significant reductions in racial disparities between the 2 arms for glucose control, modeled using the time×treatment×race interaction term. Mean glucose has an expected SD of 10.8 and distribution approximates Gaussian; thus, meeting normality assumptions. To account for dropouts and censoring events, we will increase each arm sample size to 95, totaling 190 participants. This is an inflation of approximately 10%, which is significantly above the expected totals for dropouts (estimated to be between 2% and 3%), and power reductions due to censoring events.

For aim 4, this study has adequate power to detect a statistically significant difference between the intervention arm and the national contemporary Veterans Affairs (VA) cohort for graft survival. Based on our previous research using the national VA database, we expect to identify roughly 8500 kidney transplants from the VA transplanted between 2015 and 2021, representing a contemporary cohort (33% of which are expected to be African American). Annual graft loss attrition rates are expected to be 5% in the VA cohort (with a mean of 7-year follow-up time) and 3% in the RCT treatment arm (with 2-year follow-up time). Given these, we will have 0.780 power to detect a significant difference in graft survival between the VA national cohort and our intervention patients. To assess if the intervention reduced racial disparities in graft loss, we would compare the African American persons in the RCT treatment arm to the roughly 2800 African American persons in the national VA cohort. We expect annual graft attrition rates to be 7% in the VA cohort (7-year mean follow-up) and 4% in the treatment arm (2-year follow-up). Given these estimates, we have 0.812 power to assess if the treatment intervention significantly reduced racial disparities for graft survival, as compared with the national contemporary VA cohort. These power analyses are valid across different data distribution assumptions (Gaussian and log-transformed). Power analyses were conducted using SAS 9.4 (SAS Proc GLMPower for aims 1-2; SAS Proc Power; two-sample survival test=log rank for aim 4; [SAS Institute]).

### Intervention

**Figure 1:**
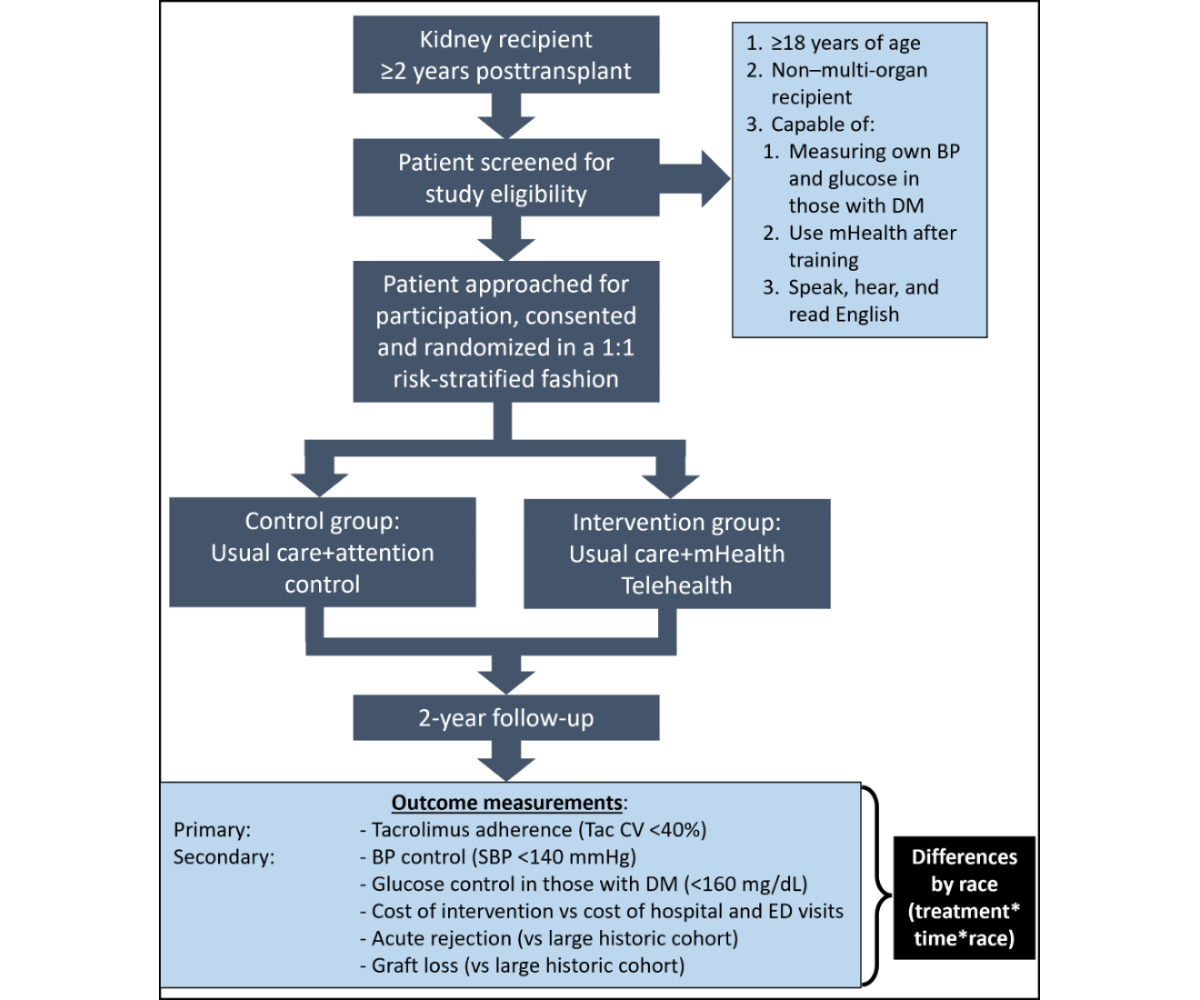
Flow of study procedures.

Patients randomized to the intervention arm will be provided the same usual care as the control group. In addition, these participants will receive comprehensive supplemental remote monitoring and follow-up by using our smartphone-enabled mHealth app and dashboard, integrated with home-based monitoring of BPs and glucoses, and pharmacist-led scheduled televisits Figure 1. Participants in this group will be provided with a smartphone and data plan if they are not current owners of a device that is compatible with the mHealth app (iOS [Apple Inc] or Android [Google]). All will also be provided with a Bluetooth-enabled, automated, cuff-style bicep home BP monitor. Those with diabetes will be provided a Bluetooth-enabled glucometer with testing supplies. On the mobile device, our mHealth app will be installed which displays the patient’s medication list and alerts them when it is time to take each medication, requiring them to respond by a push button when they have taken the specified medications, providing a time stamp of intake as part of a multi-method adherence tracking system [14]. The intervention will include pharmacist-led telemonitoring of patient adherence to medications, appointments, BP measures, glucose readings in DMs, and electronic health record (EHR) information (tacrolimus variability, appointments, and insurance status) and 21 scheduled telehealth visits with patients that cover specific topics.

#### Telehealth Visits, Activities, and Schedule

We have a detailed plan that we developed from previous work, which we will use for televisits in this study [14,20]. The pharmacists will first introduce themselves and provide a synopsis regarding the call rationale. Following this, they will conduct medication reconciliation, discuss lifestyle and diet, and review home measures to determine causes of suboptimal adherence and BP and DM control, if applicable. MI will be used to develop and implement a patient-centered plan to address identified issues leading to suboptimal control of BP, DM, and medication nonadherence. The MI follows a six-step process: (1) establishing rapport; (2) assessing knowledge, health literacy, motivation, and confidence; (3) defining barriers, concerns, and positive self-motivational statements about their behaviors; (4) summarizing “pros” and “cons” of proposed interventions to address behaviors; (5) providing options to help with adherence; and (6) giving a summary of the session, having the patient repeat back key details. During calls, the pharmacist will implement agreed-upon changes to medications and monitoring plans. This process was purposely developed so that it can be entirely conducted during televisits [24-27]. Each televisit will have a predominant theme, such as BP management, DM management, adherence, lifestyle, etc. During these 21 visits (Table 1), we will consistently use MI to better understand patient’s ambivalence toward specific issues and their intrinsic motivations and values to resolve these self-contradictory factors. Culturally competent MI will be used to work collaboratively toward goals and as a means toward improved overall well-being and health. MI is well-known to our investigator team, as we have used these in our formative research and demonstrated successful outcomes [14,16,20]. The already developed and tested mHealth and remote monitoring dashboard are supplemental technology-enabled tools that will help reinforce the telehealth sessions and provide the patient and the team with objective comprehensive data to gauge successes and continued barriers. All encounters will be documented in the EHR [14,20]. As this intervention is intense, we will also track and report back intervention delivery fidelity, call adherence, and dropout rates.

#### Smartphone mHealth App

Through an iterative, patient-centered process, we developed, tested, and validated an mHealth app that will be used within this study [14-18]. This app was used for a recently completed RCT demonstrating efficacy in reducing medication errors and improving adherence (NCT03247322) [20]. The app performed well and the feedback from enrollees is that it was well received and is useful to help with medication adherence and comorbidity self-monitoring and regulation [20]. A brief description of the app and function is further described. After a patient logs into the app using a HIPAA (Health Insurance Portability and Accountability Act)–compliant PIN (personal identification number), they are taken to their home screen. From here, they can record and review medications, BPs, glucoses, and medication side effects. They can also directly call the transplant center, study coordinator, or pharmacy for refills and send real-time email alerts to the clinician if they need to document a medication change, a visit to the ED, or admission to the hospital. When the patient taps the medications button, they can review their complete medication list, which is automatically updated from the EHR. Patients can review their medication regimen scheduled times and select which medications they are taking to document adherence. If the patient wants to document medication side effects, they tap the “Side Effects?” button, which brings them to the survey to document the incidence and severity of side effects. To review and automatically upload (using Bluetooth) BPs and blood glucoses, the patient taps the appropriate button, which brings them to the BP or glucose page. The data from Bluetooth-connected home monitoring is automatically synced to the mHealth app, encrypted, and transmitted to the web-based portal. The mHealth app also provides several important and timely push notifications to patients, including when it is time to take their medications, check their BP or glucose, and take a survey. The push notifications have a snooze function as well (every 30 minutes, up to 3 snoozes). The app provides daily individualized motivational text messages to patients, based on the self-determination theory and a comprehensive survey the patient completes at initiation [18]. These messages are automated based on how well the patient is adhering to the medication regimen and monitoring. We will enhance the app by making it compatible on Android (currently only on iOS), improve the medication data from the EHR to make it real-time (currently on an overnight delay), and add surveys.

#### Remote Monitoring Dashboard

We will also use a web-based dashboard portal that was developed as part of the aforementioned RCT [20]. The system curates data from the EHR and app and presents it in summary form on a single screen, where the clinician user can efficiently review patients (each row is a single patient), sort relevant measures (each column), and identify and triage at-risk patients. Each patient row is color-coded based on risk factors. Blue demonstrates a patient with all measures within goal, yellow demonstrates a patient with 1 measure out of range, while red indicates 2 or more measures outside targets. The thresholds for out-of-range values were set based on validated levels from previous research [7-9,11]. From this portal, the clinician can also edit patient contact information, change the passwords or login credentials for the app, update the medication regimen timing, update the timing of notifications to check home measurements, and directly text patients. Several different reporting capabilities are available through the system; reports display trends in BPs, glucoses, and medication adherence. These data can be downloaded as raw values into a spreadsheet and shared with patients or outside providers.

#### Enhanced Usual Care Control Arm

The standard care that is provided to all kidney transplant recipients will continue to be provided to both arms in this study. The structure and processes involved within this care model are well established, as evidenced by our institution’s ranking among the top-performing US transplant centers for length of stay, readmissions, and outcomes. Our kidney program was bestowed the American Society of Health-System Pharmacists Foundation Award for Excellence in Medication-Use Safety for demonstrating improvements in outcomes through a pharmacist-led, comprehensive multidisciplinary quality improvement initiative. As part of this initiative, specific protocols, which delineate immunosuppressant regimens, laboratory evaluations and timing, follow-up clinic schedules, and the treatment of comorbidities (hypertension, diabetes, and hyperlipidemia), will continue to be used. During the long-term ambulatory care phase, patients are followed with serial laboratories and clinic visits as follows. From months 6 to 12 after transplant, patients have laboratories every month with clinic visits every 3 months. From year 1 to 3 after transplant, patients are seen every 6 months with laboratories every 2 to 3 months. After year 3, patients are seen annually with laboratories every 3 months. For the enhanced usual care arm, to ensure we have home BP and glucose measure results to compare between arms, patients will be given access to the app to use in a passive mode, meaning that data will be collected, but no interventions will be delivered. Within this usual care arm, no alerts or alarms will be set within the app. If a patient does not have a compatible smartphone and data plan, one will be provided. All will be provided a Bluetooth-enabled BP device and glucometer (for DMs) and supplies. To minimize attention control bias, we will provide enhanced attention control as participants in the control group will receive SMS text messages every 7 days on general health-related topics. These SMS text messages include healthy lifestyle tips related to physical activity, dietary intake, nonexposure to first- or second-hand smoke, and limited alcohol intake.

### Safety Monitoring

The data safety monitoring plan (DSMP) will include the use of a safety officer and the MUSC Institutional Review Board to monitor study-related safety, clinical outcomes, and potential adverse events. In addition, the DSMP will use the study statistician to review the data generated by the MITIGAAT study, ensure data integrity, and assess the potential for futility. Summaries of adverse event reports and safety concerns raised by the safety officer will be made to the NIH in yearly progress reports unless the nature of a particular event is such that it bears reporting to the NIH immediately. The designated safety officer for the MITIGAAT study is a well-experienced transplant physician who is not directly involved in the intervention component of the study. The designated statistician responsible for data oversight and creating the reports needed for the DSMP meetings is an experienced biostatistician with knowledge in monitoring clinical research data integrity. Both the safety officer and the biostatistician will coordinate data review and analysis and communicate with the study principal investigator and the coinvestigators. The functions of the designated safety officer are to (1) provide scientific oversight, (2) review all serious adverse effects or complications related to the study, (3) monitor accrual, (4) review summary reports relating to compliance with protocol requirements, and (5) provide advice on resource allocation.

### Ethical Considerations

This study was submitted to the MUSC Institutional Review Board (IRB) for a full board review. The IRB reviewed and approved the protocol and reviewed consent forms to ensured protection of patient privacy and safety. The study was approved on April 18, 2023, under ID Pro00127666. Potential participants provide informed consent before participation and can opt out at any time. In order to protect participants against any risk regarding loss of personal information, all obligations under the HIPPA will be met. Any data or information shared for dissemination will be deidentified and the confidentiality of all participants will be strictly maintained. The only persons with access to protected health information will include study investigators, research coordinators, and those approved by the IRB of record. All data will be secured on university servers, behind firewalls, with passwords protecting entry in these systems. All protected health information will be obtained and managed in accordance with the HIPAA Privacy Rule (45 CFR Parts 160 and 164).

All participants will be compensated US $120 for their data use.

### Statistical Analysis Plan

For the primary outcome (aim 1, tacrolimus variability), repeated measures analyses will be conducted using generalized linear mixed modeling (GLMM). We will assess for tac CV distribution and use the appropriate link function and data transformation, if necessary, to ensure model assumptions are not violated. GLMM is the ideal model to use for this clinical trial as it provides robust estimates, takes full advantage of longitudinal data captured during the entire 2-year study, and accounts for a wide array of continuous data distribution types (eg, Gaussian, log-normal, exponential, and gamma) and discrete data distribution types for secondary outcomes (eg, Poisson, binomial, and negative binomial). Assessments for tacrolimus variability will be made every 3 months, aligning with when laboratories are typically drawn. For treatment effect estimates, both unadjusted (raw) and multivariable adjusted modeling will be conducted in an iterative manner. The time×treatment interaction term will serve as the primary measure of efficacy, which estimates the longitudinal trajectory difference between arms over the entire 2-year study. A time×treatment×race 3-way interaction term will be used to assess for changes in racial disparities, as this term estimates if the longitudinal trajectory changes between arms differ by race. Statistical significance for this term indicates that the treatment effect significantly differs by race (African American vs non–African American).

For secondary outcomes, which include BP and glucose (in those with diabetes), data will be aggregated into 25 repeated measure assessments (baseline and monthly for 2-year study). GLMM methodology will also be used for these outcome analyses. The primary efficacy measure for all secondary outcomes will be the time×treatment interaction term estimates (measure of difference in the slope between arms) from GLMM models. To specifically test for the impact of the intervention on racial disparities, the 3-way (time×treatment×race) interaction term will again be used. For both the primary and secondary outcomes, graft loss and death will be considered censoring events, and analyses will include patient data accrued up until these events occurred. To assess for differences in infection rates by treatment arm and race, Poisson or negative binomial modeling will be conducted. Model estimates for rate ratios and 95% CI will be reported. Acute rejection, graft loss, and death will be reported using Kaplan-Meier survival curves and compared using the Log Rank test. Multivariable modeling will be used to adjust for confounders, using frailty Cox regression analysis. Hazard ratios and 95% CIs will be reported. The proportional hazards assumption is tested using Schoenfeld residual plots, log (−log) plots, and *ASSESS PH* in SAS.

### Missing Data Assessment

Given we are capturing a large amount of patient-generated data, missingness may be an issue and we have devised a comprehensive plan to assess and address this. First, we will have all patients in both arms use a smartphone enabled with our mHealth app to capture and automatically send home-based, patient-generated data (BPs, glucoses, and adherence). If the patient fails to send this data, the study coordinator will reach out to the patient and facilitate capture. Second, we will use the study coordinator to conduct manual chart abstraction to reduce missing data for key baseline variables and outcomes (graft loss, death, and acute rejection). Following this, if missing data for any variable is >5%, we will conduct multiple imputation and maximum likelihood methods to address missingness. We will use fully conditional multiple imputation regression, with 20 imputations for each missing value. For outcome assessments in all aims, both unadjusted and adjusted modeling will be performed on each imputation and *PROC MIANALYZE* will be used for pooling model outputs to obtain estimates, 95% CIs, and *P* values. We will compare estimates generated from complete-case and multiple imputation analysis. To assess for missing not at random, we will implement sensitivity analysis using the missing not at random tool of *PROC* multiple imputation. We will report both sets of results (complete case and imputation results) in papers with implications addressed in the discussion [32].

### Outcomes and Definitions

The aims of this study are to improve adherence and control of late clinical issues that are predominant factors for racial disparities in kidney recipients, through a technology-enabled, telehealth-delivered, 4-level intervention. The key clinical issues for this study include tacrolimus variability, BP, and glucose control (in those with DM). We will also assess the impact on the intervention on health care use (hospitalizations and ED visits) and conduct a cost-benefit analysis. Finally, we will assess the impact of the intervention on acute rejection and graft survival rates versus a large contemporary national cohort. The study design and intervention were developed using our formative research, providing insights into interventions that change behaviors, improve access, and address provider bias and structural racism, while being highly acceptable to patients [7-9,11-21].

### Study End Points

#### Primary Outcome, Aim 1: Tacrolimus Variability

It is defined as the intrapatient tac CV; SD divided by the mean for each patient. All outpatient true trough tacrolimus levels drawn will be used to calculate the tac CV. We will assess every 3 months, which aligns with the minimum laboratory draw schedule for recipients of kidney transplant at out center. This is the sole primary outcome and will be analyzed using repeated measures methodology, estimating efficacy effect size using the time×treatment interaction term and disparity using the time×treatment×race interaction term.

#### Secondary Outcome, Aim 1

##### Time in Therapeutic Range

Time in therapeutic range (TITR) is defined as the proportion of time the tacrolimus levels for a given patient are within desired therapeutic range, typically 6-10 mg/mL. Goal ranges may vary by patient but is well documented in the medical record. TITR will be calculated using a modified version of the linear extrapolation Rosendaal method, as proposed by Reiffel [33]. All outpatient trough tacrolimus levels will be used to calculate the TITR. We will aggregate these and assess every 3 months; analyzing using repeated measures.

##### Medication Adherence Survey

It is defined based on a self-reported questionnaire administered to patients in both arms through the app every 3 months (9 total, including baseline). The Identification of Medication Adherence Barriers Questionnaire (IMAB-Q)-10 will be used; it is a validated, easy to administer, 10-question instrument. A score of <20 (range 10 to 50) indicates medication adherence.

#### Secondary Outcome, Aim 2

##### BP Assessments

It is defined as the mean of all SBPs checked by patients at home and the transplant center (ambulatory measures). Patients with a mean of SBP ≤140 mm Hg will be considered controlled. All patients will use the same home-based device for these measures and be taught proper techniques.

##### Glucose Assessments

This will only be assessed in those with a diagnosis of diabetes, estimated to be in more than 50% of the study population. Glucose control will be defined as the mean measure of all glucoses (random or fasting). Only those with diabetes will be included in this outcome. Those with DM and a mean random glucose ≤160 mg/dL will be considered as controlled. All patients will use the same meter and strips for these measures.

#### Secondary Outcome, Aim 3

##### Health Care Use

It is defined as any hospitalization or ED visit that occurs during the study period. Assessments will be analyzed using count data modeled using Poisson or negative binomial link. Total length of stay will also be assessed.

##### Hospitalizations

It is defined as any admission to a hospital with at least 1 overnight stay. Hospitalizations that occur outside the study institution will be gathered at the end of the study by querying the South Carolina Revenue and Fiscal Affairs Office, which tracks all hospitalizations for South Carolinians, regardless of payer.

##### ED Visits

It is defined as any visit to the ED with a documented encounter during the study period. ED visits that occur outside the study institution will be gathered at the end of the study by querying the South Carolina Revenue and Fiscal Affairs Office, which tracks all ED visits.

#### Aim 4

##### Acute Rejection

It is defined as a renal allograft biopsy showing at least grade 1A rejection by Banff criteria [34,35]. As per usual care practices, all patients are required to have biopsy confirmation of rejection episodes within 24 hours of onset of treatment for acute rejection. It is standard care that all kidney allograft biopsies performed for recipients of transplant occur at the transplant center (study institution). Biopsies will be read by a blinded local pathologist, as usual care. This will be assessed using time to event analyses.

##### Graft Failure and Death

Graft failure will be defined as return to chronic dialysis, nephrectomy, retransplant, or death. The timing and cause of each graft loss will be recorded for comparative analysis. Patient death will also be captured, with timing and cause. These will be analyzed using time to event methodology. For primary and secondary outcomes, graft loss and death will be considered censoring events; data accrued until these events will be used in analyses.

## Results

This study was funded in July 2023. The first patient was enrolled in April 2024, and the study is 25% (49/190) enrolled. We expect to complete enrollment in 2026. All patients will complete the study by the end of 2028, and results can be expected shortly after.

## Discussion

We propose an RCT to use this system to deliberately address posttransplant disparities in African American people, entitled the MITIGAAT study. The overarching hypothesis for the MITIGAAT study is that late nonadherence and suboptimal control of diabetes and hypertension are more common in African American kidney recipients and are major contributors to health disparities. A multimodal intervention that addresses these issues should significantly reduce health care disparities among African American recipients of kidney transplant.

## Abbreviations

AHRQ: Agency for Healthcare Research and Quality
BP: blood pressure
DM: diabetes mellitus
DSMP: data safety monitoring plan
ED: emergency department
EHR: electronic health record
GLMM: generalized linear mixed modeling
HIPAA: Health Insurance Portability and Accountability Act
IMAB-Q: Identification of Medication Adherence Barriers Questionnaire
IRB: Institutional Review Board
mHealth: mobile health
MI: motivational interviewing
MITIGAAT: Multifaceted Intervention to Improve Graft Outcome Disparities in African American Kidney Transplants
MUSC: Medical University of South Carolina
NIDDK: National Institute of Diabetes and Digestive and Kidney Diseases
NIH: National Institute of Health
PIN: personal identification number
RCT: randomized controlled trial
REDCap: Research Electronic Data Capture
SBP: systolic blood pressure
tac CV: tacrolimus concentration coefficient of variation
TITR: time in therapeutic range
VA: Veterans Affairs
